# Genetic characterization of the respiratory tract viruses in Jilin, Northeast China, 2023

**DOI:** 10.3389/fpubh.2025.1756127

**Published:** 2026-01-13

**Authors:** Zhixia Song, Yuanyuan Huang, Yue Gu, Lihe Che, Kaiyu Zhang, Quan Liu, Qingtian Guan, Liyan Sui

**Affiliations:** 1 State Key Laboratory for Diagnosis and Treatment of Severe Zoonotic Infectious Diseases, Department of Infectious Diseases and Center of Infectious Diseases and Pathogen Biology, Key Laboratory of Organ Regeneration and Transplantation of the Ministry of Education, The First Hospital of Jilin University, Changchun, China; 2 Department of Paediatrics, First Hospital of Jilin University, Changchun, China; 3 Department of Respiratory and Critical Care Medicine, First Hospital of Jilin University, Changchun, China; 4 Bioinformatics Laboratory, Infectious Diseases and Pathogen Biology Center, The First Hospital of Jilin University, Changchun, China

**Keywords:** human pegivirus, metagenomic sequencing, novel picobirnaviru*s*, phylogenetic analyses, respiratory viruses

## Abstract

**Objective:**

Respiratory viral infections impose a significant global health burden, necessitating continuous regional surveillance to understand pathogen circulation. This study aimed to characterize the spectrum of respiratory pathogens and identify potential causative agents in Jilin Province, northeast China, during 2023.

**Methods:**

Using metagenomic next-generation sequencing, we analyzed 250 respiratory samples and 195 blood samples, sequencing of all samples yielded 399,256 viral reads. Bioinformatic and phylogenetic analyses were conducted to identify and characterize the detected viruses.

**Results:**

Severe acute respiratory syndrome coronavirus 2 (lineage BA.2), human respiratory syncytial virus B (lineage GB5.0.5a), and influenza B virus (lineage V1A.3a.2) were identified as common respiratory pathogens across both pediatric and adult populations. Influenza A virus (lineage 3C.2a1b.2a.2a.3a.1), rhinovirus (subtype C), human respiratory syncytial virus A (lineage GA2.3.5), human respiratory syncytial virus B (lineage GB5.0.5a), and human metapneumovirus (lineage A2c) were detected in pediatric or adult respiratory samples. Human Pegivirus (genotype 3) was detected exclusively in adult blood samples. Strikingly, a novel picobirnavirus was identified in adult sputum samples, sequence and structural analyses consistently indicate that this picobirnavirus is closely related to human-associated strains, exhibiting ≥70% amino acid identity and an RdRP structure nearly identical to that of picobirnaviruses previously identified in human upper respiratory swabs from Cambodia. This finding was validated by nested RT-PCR, representing the first documented detection of picobirnavirus in respiratory specimens from China. As most identified strains were first reported in northeast China, we also conducted comprehensive phylogenetic analyses of representative viruses, revealing high sequence similarity with epidemic strains from other regions of China.

**Conclusion:**

These findings delineate the respiratory viruses of northeast China, providing data for region-specific surveillance to mitigate future public health risks.

## Introduction

Respiratory viral infections present a persistent and severe threat to global health, which rank among the most prevalent human illnesses worldwide, with RNA viruses dominating the spectrum of respiratory pathogens (1). Key agents of concern include influenza viruses (IV), human respiratory syncytial virus (HRSV), human rhinoviruses (HRV), and coronaviruses (CoVs). Notably, HRSV is the leading cause of severe lower respiratory tract infections (e.g., bronchiolitis) and mortality in infants under one year of age (2). Influenza A virus (IAV) remains a major public health challenge, having triggered devastating pandemics in 1918, 1957, and 1968 that collectively claimed millions of lives (3, 4). Advances in scientific research and healthcare infrastructure over recent decades have mitigated mortality linked to respiratory viruses, including IAV (H1N1) pdm09 and *betacoronaviruses* such as SARS-CoV and MERS-CoV (5, 6). However, the emergence of SARS-CoV-2, the novel *betacoronavirus* responsible for COVID-19, in late 2019 abruptly reversed this progress, plunging the world into an unprecedented health crisis marked by widespread morbidity and economic disruption. *Severe Acute* Respiratory Syndrome Coronavirus 2 (SARS-CoV-2) has since transitioned from a pandemic threat to an endemic pathogen, joining seasonal *coronaviruses* as a recurrent challenge to global health (Johns Hopkins Coronavirus Resource Center, 2022).

In 2023, influenza was the leading viral cause of lower respiratory infections (excluding COVID-19), accounting for an estimated 22.5 million (19.6–26.5) episodes globally (7). Respiratory syncytial virus (RSV) remains a major cause of pediatric hospitalizations worldwide; among children under 5 years, its incidence rate reached 1187.5 (972.0–1433.6) per 100,000 in 2023 (7, 8). This pattern is corroborated by a large-scale etiological and epidemiological analysis of 231,107 patients in China, which identified influenza virus (28.5%), RSV (16.8%), and human rhinovirus (16.7%) as the three most prevalent viral pathogens (9). Together, these findings underscore the substantial global impact of respiratory viruses and emphasize the need for data-driven prevention, vaccine deployment, and equitable health interventions. Comprehensively cataloging viruses present in the respiratory tract is critical for understanding their role in infections and disease progression. Conventional diagnostic approaches frequently fall short in identifying the full spectrum of viral pathogens or delivering conclusive diagnoses, hindering effective responses to emerging, evolving, or rare viral strains. In recent years, metagenomic next-generation sequencing (mNGS) has transformed viral discovery by enabling unbiased pathogen detection without requiring prior genetic information about target organisms (10, 11). Human-associated viruses, including those colonizing the gut, respiratory tract, and bloodstream, have been extensively characterized through mNGS, revealing novel viral lineages and host-pathogen dynamics (12–16). The northeast region of China, characterized by its cold climate, high population density in urban areas, and distinct seasonal variations, presents a unique ecological niche for respiratory viruses. However, this region has been scarcely studied for these pathogens. Here, we applied mNGS framework to systematically profile both known and emerging viral pathogens associated with respiratory infections in patients from Jilin Province, northeast China. This approach aims to enhance detection of understudied or divergent viral strains, which provides a foundation for region-specific pathogen surveillance and enables data-driven public health strategies.

## Results

### Overview of sequenced libraries and detected pathogen in reads

**Figure 1:**
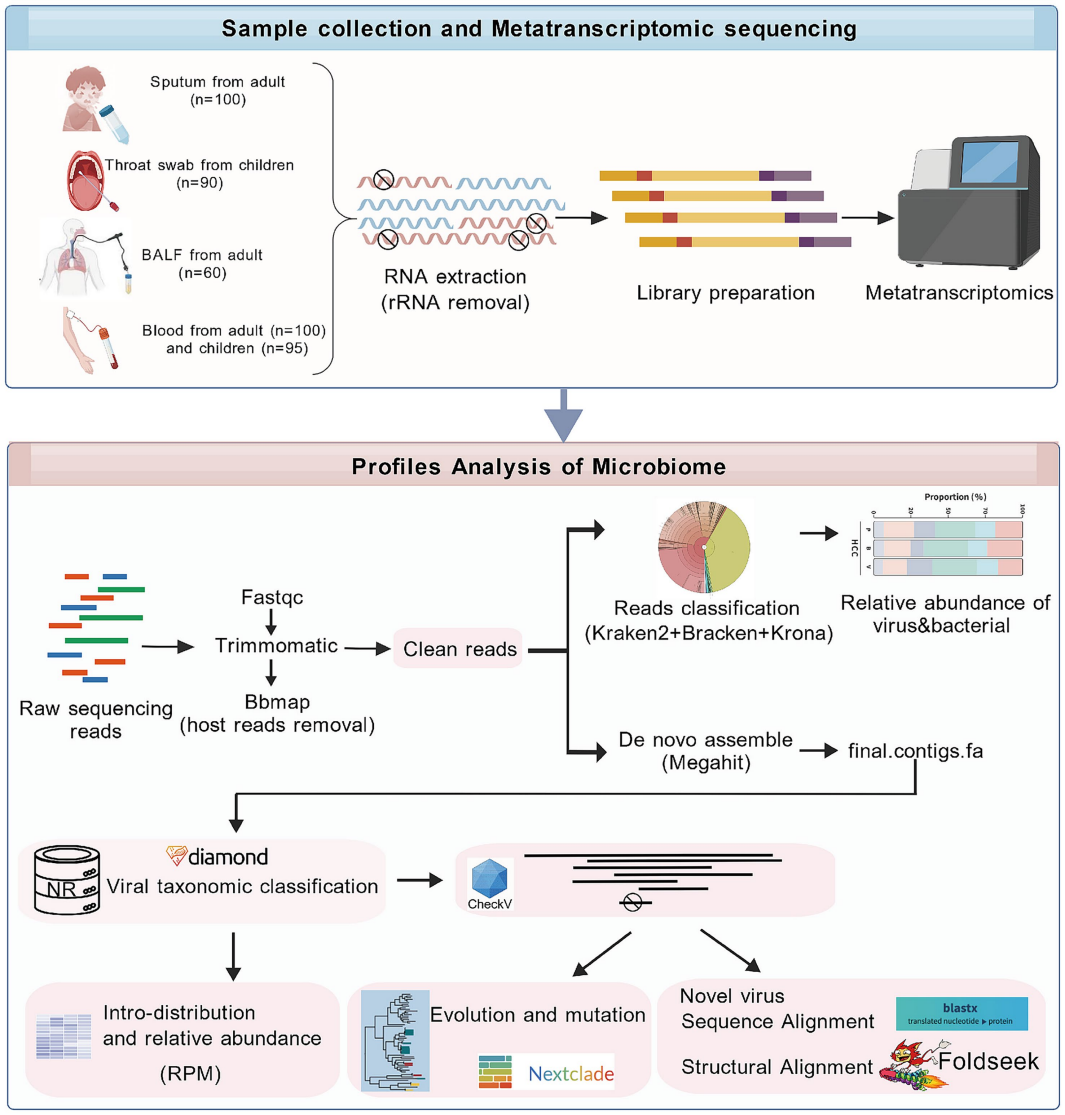
Metatranscriptomics workflow. The illustration was created with BioGDP.com.

**Figure 2:**
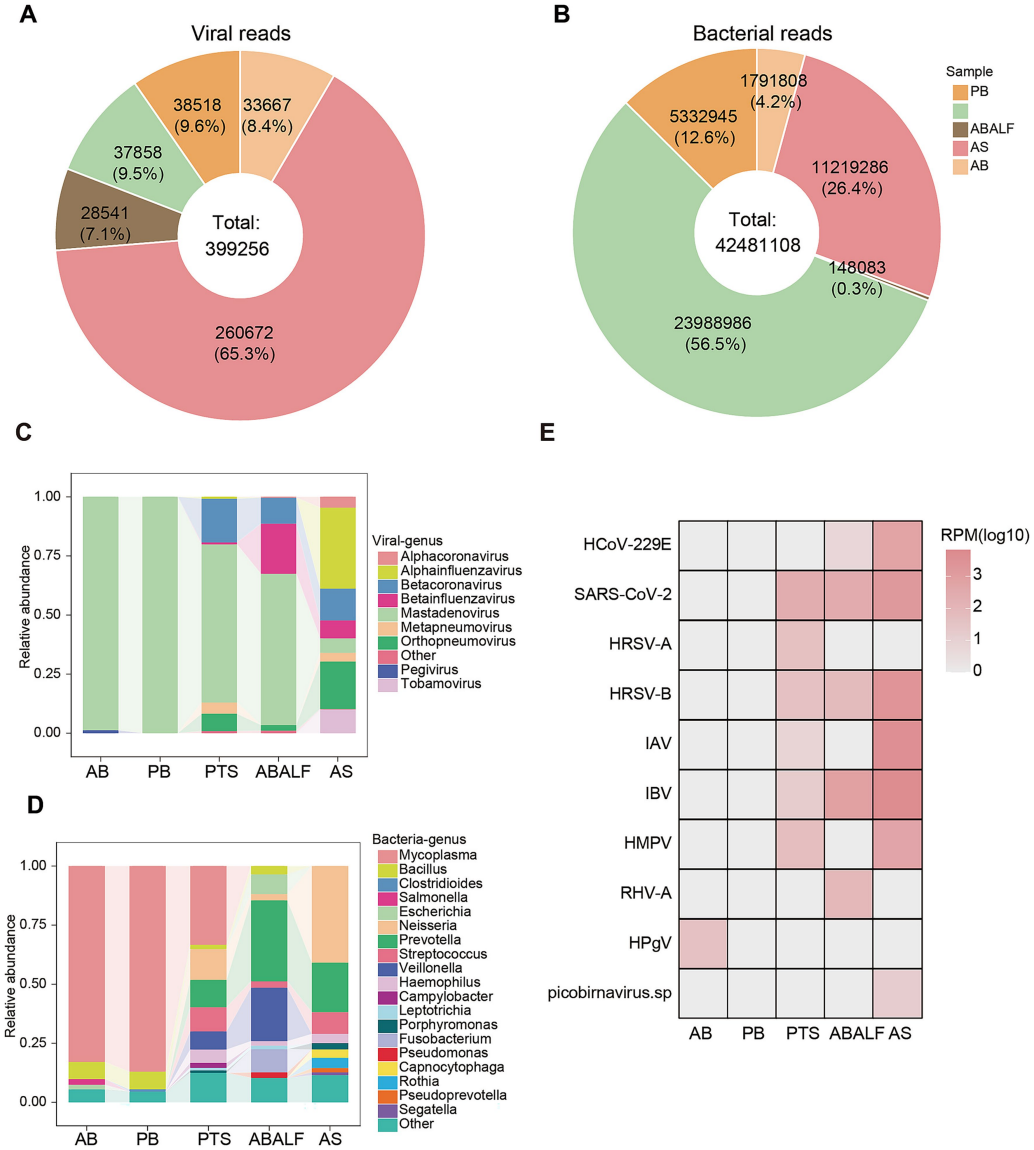
Taxonomic composition and distribution of microbial communities across clinical specimens **(A,B)** Comparative analysis of viral **(A)** and bacterial **(B)** read proportions and absolute counts in five clinical sample types: pediatric blood (PB), pediatric throat swab (PTS), adult bronchoalveolar lavage fluid (ABALF), adult sputum (AS), and adult blood (AB). **(C,D)** Relative abundance of viral genus **(C)** and bacterial genus **(D)** in each of libraries. **(E)** Distribution and abundance of viruses in all type of clinical samples. The relative abundance of viruses in each library was calculated and normalized by the number of mapped reads per million total reads (RPM).

We characterized the viromes of 445 samples collected from Jilin Province, northeast China. Among these, 90 samples were pediatric throat swabs (PTS) and 95 were pediatric blood (PB) samples, with all pediatric patients aged ≤14 years. Notably, 60 of these samples were collected as matched pairs, meaning that both a throat swab and a blood sample were obtained from the same individual patient. The remaining samples were obtained from different individuals. Additionally, there were 100 adult blood (AB) samples, 100 adult sputum (AS) samples, and 60 adult bronchoalveolar lavage fluid (ABALF) samples. Samples were pooled into five categories (PB, PTS, ABALF, AS, AB) based on type and age group prior to RNA extraction (Figure 1). High-throughput sequencing generated a total of 101,037,128 paired-end reads. Taxonomic classification of the reads was performed using Kraken2, which classified 399,256 paired-end reads as virus, with 42,481,108 reads assigned as bacterial (Figures 2A,B).

Schematic overview of metatranscriptomics sample preparation and data analysis. Total RNA was extracted from clinical samples, and human rRNA was depleted before library preparation and sequencing. After adapter trimming and host sequence removal of the raw sequencing data, the remaining reads were classified and assembled. The resulting contigs were aligned against the NCBI nr database using diamond to identify virus-associated sequences, followed by analyses of viral community abundance, phylogeny, and structural and sequence comparisons of newly discovered viruses.

Viral and bacterial compositions were quantitatively analyzed at the genus level across sample types. After filtering taxa with relative abundances below 0.01, spanning 9 viral generas were retained, representing *Adenoviridae*, *Coronaviridae, Orthomyxoviridae, Pneumoviridae,* and *Flaviviridae* families. Viral read distribution exhibited significant sample-type specificity: respiratory specimens dominated virome composition, with ≥82% of viral reads localized to respiratory samples, with adult sputum (AS) contributing 65.3% of total viral reads, whereas clinical blood samples showed minimal representation (18%), confirming respiratory tract as the primary viral niche (Figure 2A). Notably, bronchoalveolar lavage fluid (ABALF) samples exhibited the lowest proportion of viral reads among respiratory specimens, likely reflecting their origin from the lower respiratory tract, where viral loads are generally lower. Distinct viral distribution patterns emerged across cohorts. *Mastadenovirus* was ubiquitous among all 5 pools, maintaining near-complete dominance (relative abundance ≈1) in both adult and pediatric blood specimens. *Pegivirus* displayed adult blood-specific colonization, absent in pediatric counterparts. While respiratory-restricted viruses (*Orthopneumovirus*, *Betacoronavirus, Betainfluenzavirus*) consistently populated in all respiratory matrices (adult bronchoalveolar lavage fluid, sputum and pediatric throat swabs) (Figure 2C).

Bacterial profiling identified a total of 19 genera from 5 sample pools. Blood specimens exhibited the lowest microbial diversity, with distinct taxonomic signatures between adult and pediatric cohorts. *Mycoplasma* (≥80% total abundance) and *Bacillus* were detected in both adult and pediatric blood samples (Figure 2D). *Salmonella* and *Escherichia* were exclusively detected in adult blood samples, whereas *Clostridioides* was uniquely present in pediatric blood. Similarly, *Campylobacter* was exclusively found in pediatric throat swabs but not detected in the upper or lower respiratory tracts of adults. In contrast, *Pseudomonas* was only detected in the lower respiratory tract (adult bronchoalveolar lavage fluid) (Figure 2D).

### Assembled viral species and abundance

Totally, complete genomes of eight highly transmissible human respiratory viruses were assembled: SARS-CoV-2, *Human coronavirus 229E* (HCoV-229E), HRSV-A, HRSV-B, *Human metapneumovirus* (HMPV), RHV-A, IAV, and *Influenza B virus* (IBV), which showed ≥98% similarity to genomes previously uploaded to NCBI. SARS-CoV-2, HRSV-B, and IBV were present in all respiratory samples, including the upper respiratory tract of children, and both the upper and lower respiratory tracts of adults (Figure 2E). In pediatric blood samples, only partial human adenovirus genomes (coverage≤6.35%) were assembled. In adult blood samples, nearly a complete genome of Human pegivirus (HPgV) were detected, which showed a similarity of 92.76% with the sequences in NCBI (Genebank accession number: MZ099568.1) (Table 1 and Supplementary Table S1).

**Table 1:** Viral species assembled from the RNA sequencing libraries.

Viral sequences identified in samples	Sample type	Query cover	Nucleotide identity to closest sequence	Closest sequence accession number	Sequence length	Assembly length	Assembly accession number
Human coronavirus 229E	AS	100%	99.83%	ON791801.1	27,284	27,220	PV631258
Severe acute respiratory syndrome coronavirus 2	AS	100%	99.99%	PQ025106.1	29,843	29,833	PV639771
Severe acute respiratory syndrome coronavirus 2	PTS	96%	99.97%	OR722151.1	29,816	30,998	PV570237
Severe acute respiratory syndrome coronavirus 2	ABALF	100%	99.87%	OR616982.1	29,814	29,790	PV570236
Human respiratory syncytial virus A	PTS	100%	99.58%	PP833560.1	15,227	15,066	PV605107
Human respiratory syncytial virus B	PTS	100%	99.87%	PP974214.1	15,081	15,081	PV603865
Human respiratory syncytial virus B	AS	95%	99.70%	OR795238.1	15,219	15,196	PV603866
Human respiratory syncytial virus B	ABALF	100%	99.66%	OR326803.1	15,195	15,129	PV603867
Influenza A virus segment 4	AS	91%	99.88%	OY998351.1.1	1762	1730	PV571916
Influenza A virus segment 4	PTS	100%	99.65%	OR201918.1	1737	1703	PV571911
Influenza B virus segment 4	PTS	100%	99.94%	PP295217.1	1749	1,670	PV597931
Influenza B virus segment 4	AS	93%	99.89%	PQ069917.1	1847	1914	PV597950
Influenza B virus segment 4	ABALF	100%	99.78%	PP464059.1	1864	1922	PV597941
Human metapneumovirus	PTS	100%	99.24%	OR671980.1	13,435	13,354	PV611499
Human metapneumovirus	AS	100%	99.14%	MN745086.1	13,430	13,437	PV612395
Rhinovirus A	ABALF	100%	98.01%	LC789193.1	7,156	7,098	PV611497
Human pegivirus	AB	100%	92.76%	MZ099568.1	9,273	7,964	PV611498
Picobirnavirus sp. segment 1	AS	80%	91.93%	OL875303.1	2,115	2,477	PV631260
Picobirnavirus sp. segment 2	AS	3%	92.06%	PQ792594.1	1828	1826	PV631259

Nine known virus species and one novel picobirnavirus.

Query cover (%): Proportion of the assembled viral sequence that aligns to the reference genome, expressed as a percentage; PB, pediatric blood; PTS, pediatric throat swab; ABALF, adult bronchoalveolar lavage fluid; AS, adult sputum; AB, adult blood.

In addition to known human-associated viruses, we identified a novel human picobirnavirus from the adult sputum. Its genome consists of two segments: segment 1 (GenBank accession number: PV631260) encoding the capsid protein, and segment 2 (GenBank accession number: PV631259) encoding the RNA-dependent RNA polymerase (RdRp), which showed ≥90% identity with the sequences in NCBI with less than 5% genome coverage. This represents the first documented picobirnavirus in Chinese respiratory specimens.

### Evolutionary analysis

#### Coronaviridae

Two *Coronaviridae* virus species were identified from samples: *Betacoronavirus* SARS-CoV-2, detected in pediatric throat swabs and adult bronchoalveolar lavage fluid/sputum, and *Alphacoronavirus* HCoV-229E, found in adult bronchoalveolar lavage fluid and sputum. A phylogenetic tree was constructed using the full genome sequences.

**Figure 3:**
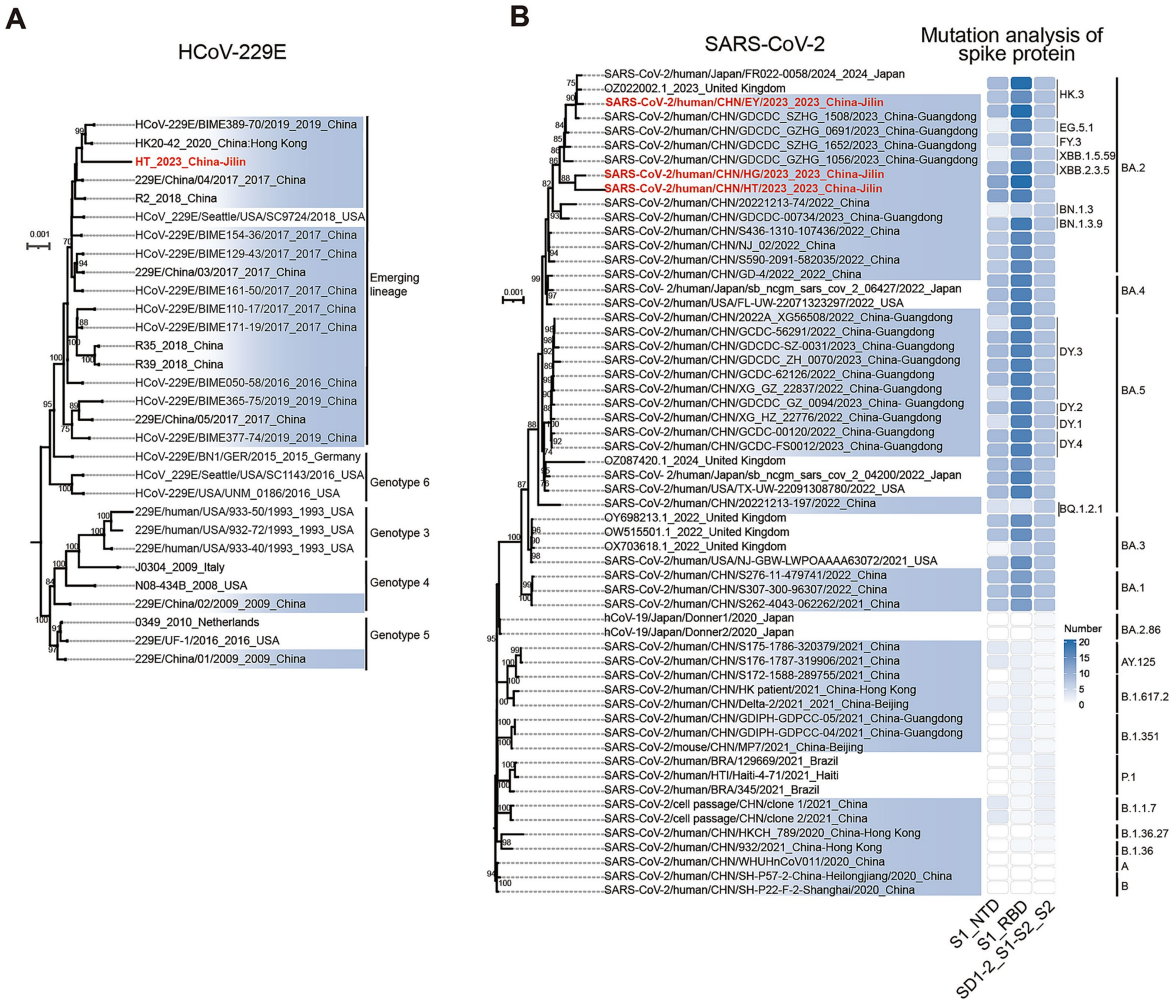
Maximum likelihood phylogenetic trees of *Coronaviridae* and analysis of spike protein variants. **(A)** Phylogenetic tree of HCoV-229E. The HCoV-229E identified in this study belongs to emerging lineage. **(B)** Phylogenetic tree of SARS-CoV-2 and variants of S protein. Using the Pangolin lineage method, the SARS-CoV-2 identified in this study was classified into the BA.2 lineage. The tree was constructed using complete genome nucleotide sequences. Sequences highlighted in blue represent genomes uploaded from China, while sequences labeled in red indicate genomes assembled in this study. In the S-protein mutation analysis, the S protein was divided into three domains according to the NCBI concise classification standard. N-terminal domain of S1 (S1_NTD): spanning amino acids 13–304. Receptor-binding domain of S1 (S1_RBD): spanning amino acids 319–541. SD-1 and SD-2 subdomains, the S1/S2 cleavage region, and the S2fusion subunit of the spike (S) glycoprotein (SD1-2_S1-S2_S2): spanning amino acids 543–1,208.

For HCoV-229E, sequences were retrieved from the National Center for Biotechnology Information (NCBI) Virus database (https://www.ncbi.nlm.nih.gov/labs/virus) using the following criteria: geographic region (China), and sequence length (≥26,000 bases to cover the full coding sequence). A total of 55 nucleotide sequences were obtained. Geographic metadata analysis revealed no complete HCoV-229E sequences from northeastern China in the dataset. Redundancy reduction was performed using CD-HIT (version 4.8.1) with a 99.8% similarity threshold, while all other parameters were set to their default values. The phylogenetic tree incorporated globally reference sequences of known lineages, deduplicated Chinese sequences, and the HCoV-229E complete sequence assembled from adult sputum samples in this study. Phylogenetic analysis demonstrated that the HCoV-229E sequence clusters within an emerging lineage and exhibits high similarity with strains previously identified in Beijing (2019), northern China (Figure 3A).

For SARS-CoV-2, lineage classification was conducted using the Pangolin framework. Reference sequences of known lineages were downloaded from the NCBI Virus database. Under identical geographic (China) and quality filters (complete genome, ≥28,000 bases), 3,172 complete SARS-CoV-2 sequences were retrieved from China, which underwent redundancy reduction via CD-HIT (99% similarity threshold). The phylogenetic tree included international reference sequences, the northeastern China sequence, deduplicated Chinese sequences, and three SARS-CoV-2 sequences generated in this study. All three sequences derived from respiratory pools were classified into the BA.2 lineage, with the pediatric throat swab sequence further resolved into the HK.3 sublineage (Figure 3B). Notably, two genomes from adult respiratory samples clustered within BA.2 but formed a distinct subclade, suggesting uncharacterized regional diversity (Figure 3B).

The spike (S) protein of SARS-CoV-2 plays a critical role in target recognition and cellular entry, thereby facilitating viral infection. Mutations in this protein have contributed to the emergence of new SARS-CoV-2 variants with significantly enhanced overall fitness. In this study, we conducted a comprehensive mutation analysis of the spike protein across all SARS-CoV-2 strains included in the phylogenetic tree. A total of 1,582 distinct mutations were identified, among which the top 50 most frequent mutations accounted for 1,223 occurrences (77.3%). Analysis of these top 50 mutations revealed that most strains exhibited the highest number of substitutions within the S1 receptor-binding domain (S1_RBD). Combined with lineage classification, we found that the Omicron/BA series (BA.1, BA.2, BA.3, BA.4, and BA.5) harbored the greatest number of S-protein mutations (Figure 3B). In the BA.2 lineage, only the SARS-CoV-2/human/CHN/HT/2023 strain identified in this study harbored the H69/V70 deletion, a mutation previously shown to enhance immune evasion and contribute to reduced S-gene target detection (17). In contrast, this deletion is present in other Omicron sublineages, including BA.1, BA.3, BA.4, and BA.5. Within the BA.2 lineage, the N211 deletion and L212I mutations were uniquely identified in the SARS-CoV-2/human/CHN/HT/2023 and SARS-CoV-2/human/CHN/HG/2023 strains. Notably, these mutations are characteristic of the BA.1 and BA.3 lineages. This shared characteristic may explain why these two strains form a distinct sublineage separate from other BA.2 strains circulating in Guangdong during 2022 and 2023. In fact, these mutations have been experimentally demonstrated to disrupt the tight binding of the peptide PINLVRDLPQGFSAL to the HLA-DRB1*03:01 molecule, resulting in the loss or substantial reduction of this HLA class II epitope’s binding affinity (S1) and potentially impairing effective T-cell immunity (18).

#### Orthopneumovirus

HRSV, a single-stranded negative-sense RNA virus within the *Pneumoviridae* family and *Orthopneumovirus* genus, is categorized into two subtypes (HRSV-A and HRSV-B), each comprising multiple genotypes. In this study, one HRSV-A sequence was assembled from pediatric throat swabs, while three HRSV-B sequences were obtained from pediatric throat swabs, adult bronchoalveolar lavage fluid, and adult sputum.

**Figure 4:**
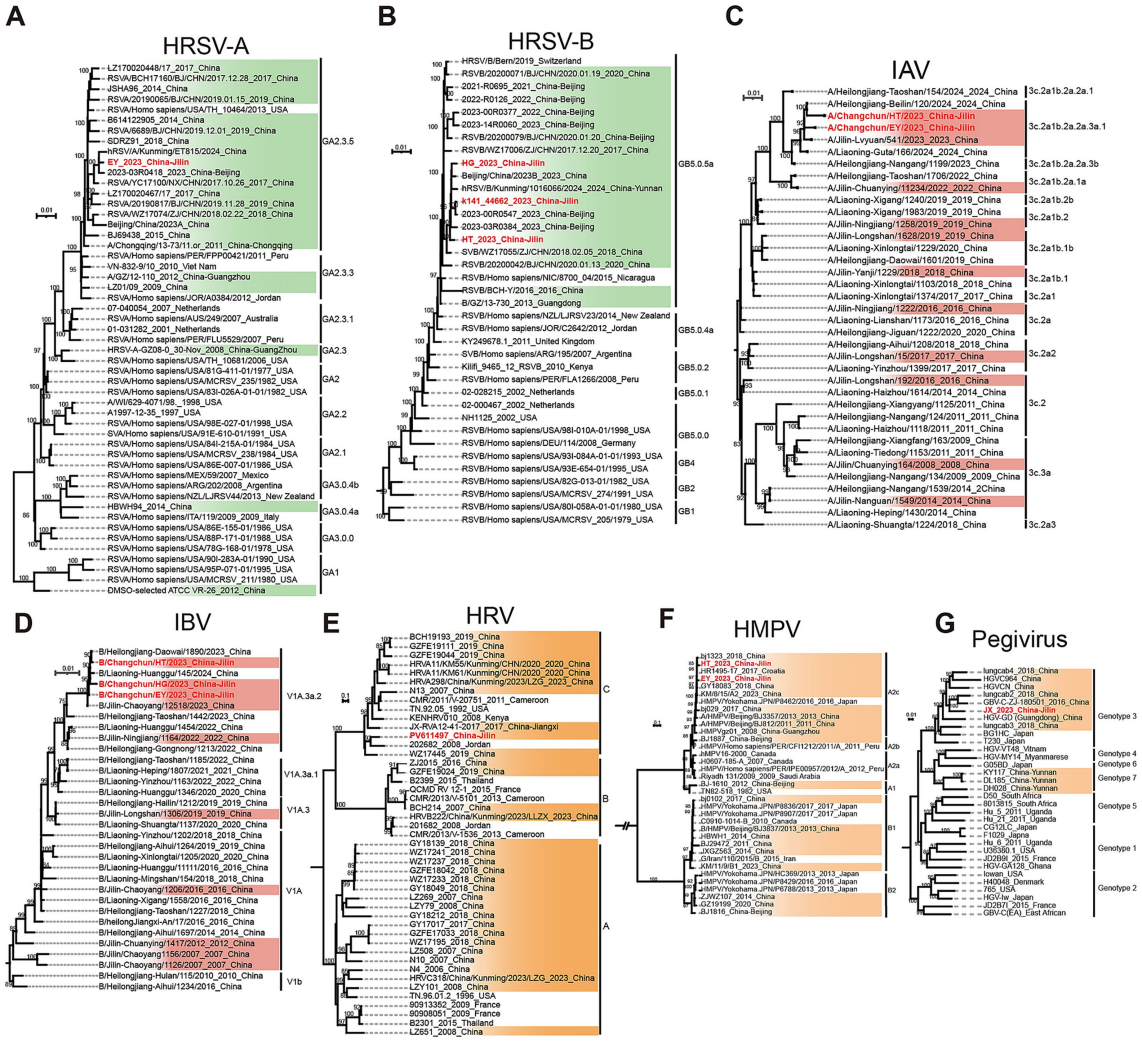
Maximum likelihood phylogenetic trees. **(A)** Phylogenetic tree of HRSV-A. The HRSV-A identified in this study belongs to GA2.3.5 branch. **(B)** Phylogenetic tree of HRSV-B. The HRSV-B identified in this study was classified into the GB5.0.5a branch. **(A,B)** The tree was constructed using complete genome nucleotide sequences. Sequences highlighted in green represent genomes uploaded from China, while sequences labeled in red indicate genomes assembled in this study. **(C)** Phylogenetic tree of IAV. The IAV identified in this study belongs to 3c.2a1b.2a.2a.3a.1 clade. **(D)** Phylogenetic tree of IBV. The IBV identified in this study was classified into the V1A.3a.2 clade. **(C,D)** The phylogenetic tree was constructed based on nucleotide sequences encoding the hemagglutinin (HA) protein (segment 4). Sequences highlighted in red represent genomes uploaded from Jilin, China; sequences labeled in red indicate genomes assembled in this study. **(E)** Phylogenetic tree of HRV. The phylogenetic tree of HRV constructed based on nucleotide sequences encoding the VP4/VP2 protein. The HRV identified in this study belongs to C subtype. **(F)** Phylogenetic tree of HMPV. The phylogenetic tree of HMPV constructed based on nucleotide sequences encoding the glycoprotein protein. The HMPV identified in this study belongs to A2c subtype. **(G)** Maximum likelihood phylogenetic tree of pegivirus. The phylogenetic tree was constructed with the complete genome nucleotide sequences. The pegivirus identified in this study belongs to genotype 3. (E–G) Sequences highlighted in orange represent genomes uploaded from China, while sequences labeled in red indicate genomes assembled in this study.

Globally reference sequences of known HRSV genotypes and all complete Chinese HRSV genomes (sequence length ≥14,000 bases) were retrieved from the NCBI database. This yielded 100 HRSV-A and 86 HRSV-B complete sequences from China. However, geographic metadata analysis revealed no sequences originating from northeastern China. Redundancy reduction was performed using CD-HIT with sequence similarity thresholds of 99.2% (HRSV-A) and 99.4% (HRSV-B). Phylogenetic trees were constructed using international reference sequences, deduplicated Chinese sequences, and the HRSV genomes assembled in this study. Phylogenetic analysis demonstrated that the HRSV-A sequence (Jilin Province, 2023) clusters within genotype GA2.3.5, showing closest homology to sequences from Kunming (2024) and Beijing (2023) (Figure 4A). All three HRSV-B sequences and the deduplicated Chinese HRSV-B genomes were classified into genotype GB5.0.5a (Figure 4B).

#### Orthomyxoviridae

IAV and IBV, members of the *Orthomyxoviridae* family (genera *Alphainfluenzavirus* and *Betainfluenzavirus*, respectively), are single-stranded negative-sense RNA viruses. Their genomes comprise eight segments encoding PB2, PB1, PA, HA, NP, NA, M, and NS proteins. In this study, IAV (subtype H3N2) was detected in pediatric throat swabs and adult sputum samples collected in Jilin Province, China (2023). Additionally, IBV was identified in pediatric throat swabs, adult bronchoalveolar lavage fluid, and adult sputum.

A total of 5,110 HA protein-coding sequences of H3N2 viruses representing all known clades from China were downloaded from GISAID. Among these, 228 sequences from 11 clades were originated from northeastern China. After redundancy reduction using CD-HIT, 35 unique sequences from northeastern China were retained. These sequences, along with two HA protein-coding sequences assembled in this study, were used to construct a phylogenetic tree. Phylogenetic analysis revealed that the IAV viruses identified in this study belong to the 3C.2a1b.2a.2a.3a.1 clade and are most closely related to sequences detected in human samples from Lvyuan District, Changchun City, Jilin Province in 2023 and from Heilongjiang Province in 2024 (Figure 4C).

For IBV, a total of 3,858 HA protein-coding sequences from the Victoria lineage representing all known clades from China were downloaded from GISAID. Of these, 155 sequences from five clades were derived from northeastern China. Following redundancy reduction with CD-HIT, 29 unique sequences were retained. These sequences, together with three HA protein-coding sequences assembled in this study were used to construct a phylogenetic tree. The analysis indicated that the IBV sequences obtained in this study were closely related to IBV sequences identified in 2023 from Chaoyang District, Changchun City, Jilin Province, in 2024 from Liaoning Province, and in 2023 from Heilongjiang Province, which belongs to the V1A.3a.2 clade (Figure 4D).

### Rhinovirus (HRV) and human metapneumovirus (HMPV)

HRV, a single-stranded positive-sense RNA virus belonging to the *Picornaviridae* family and *Enterovirus* genus, is categorized into three subtypes (A, B, and C). Phylogenetic analysis here focused on VP4/VP2 protein-coding nucleotide sequences. A complete HRV genome was assembled from adult bronchoalveolar lavage fluid. International reference sequences of known subtypes and all complete Chinese HRV genomes (≥6,000 bp) were downloaded from NCBI, yielding 40 sequences from China. Geographic metadata confirmed no HRV genomes from northeastern China. Redundancy reduction was applied using CD-HIT (99% similarity threshold). The phylogenetic tree integrated globally reference sequences, deduplicated Chinese genomes, and the HRV sequence generated in this study. Phylogenetic analysis revealed time-dependent evolution of HRV: clades A and B exclusively contained strains identified before 2020. In contrast, Clade C has been the exclusive lineage circulating since 2020, indicating a recent clade replacement. Consistent with this pattern, the HRV sequence from northeastern China (winter 2023) was classified into clade C (Figure 4E).

HMPV, a single-stranded negative-sense RNA virus within the *Pneumoviridae* family and *Metapneumovirus* genus, is classified into two genotypes (A and B) and expresses two major immunogenic surface proteins: the attachment glycoprotein (G) and fusion (F) protein. In this study, phylogenetic analysis focused on the G protein-coding region. Two complete HMPV genomes were assembled from pediatric throat swabs and adult sputum. Globally reference sequences of known genotypes and all complete Chinese HMPV genomes (≥12,000 bases) were retrieved from NCBI using geographic (China) and completeness (complete genome) yielding 79 sequences from China. Geographic metadata analysis revealed no representatives from northeastern China. Redundancy reduction was performed on the Chinese sequences using CD-HIT (98.8% similarity threshold). The strains identified at different and locations in China displayed broad distribution in both A and B clades. HMPV strains from diverse locations across China were distributed across both A and B clades. Phylogenetic analysis revealed that the HMPV sequence from this study clusters within subtype A2c, demonstrating high genetic similarity to contemporaneous strains from multiple Chinese regions, including Beijing (north), Guangzhou, and Kunming (south) (Figure 4F).

### Human pegivirus (HPgV)

HPgV, a single-stranded positive-sense RNA virus [ssRNA (+)] within the family *Flaviviridae* and genus *Pegivirus*, is classified into seven genotypes (1–7). In this study, phylogenetic analysis was performed using complete genome sequences. A nearly complete HPgV genome (nearly 8,000 bp) was assembled from an adult blood sample collected in northeastern China during winter 2023. International reference sequences representing known genotypes and all nearly complete Chinese HPgV genomes were retrieved from NCBI using geographic (China) and length (≥7,000 bp) filters, yielding 4 sequences from China. Geographic metadata analysis confirmed no prior HPgV genomes from northeastern China. A phylogenetic tree was constructed using globally reference sequences, the Chinese genomes, and the study-derived HPgV sequence. Phylogenetic analysis classified the northeastern China HPgV sequence into genotype 3 (Figure 4G). Notably, only genotypes 3 and 7 were represented among the Chinese HPgV genomes in the dataset, with no sequences from other genotypes identified.

### Picobirnavirus

**Figure 5:**
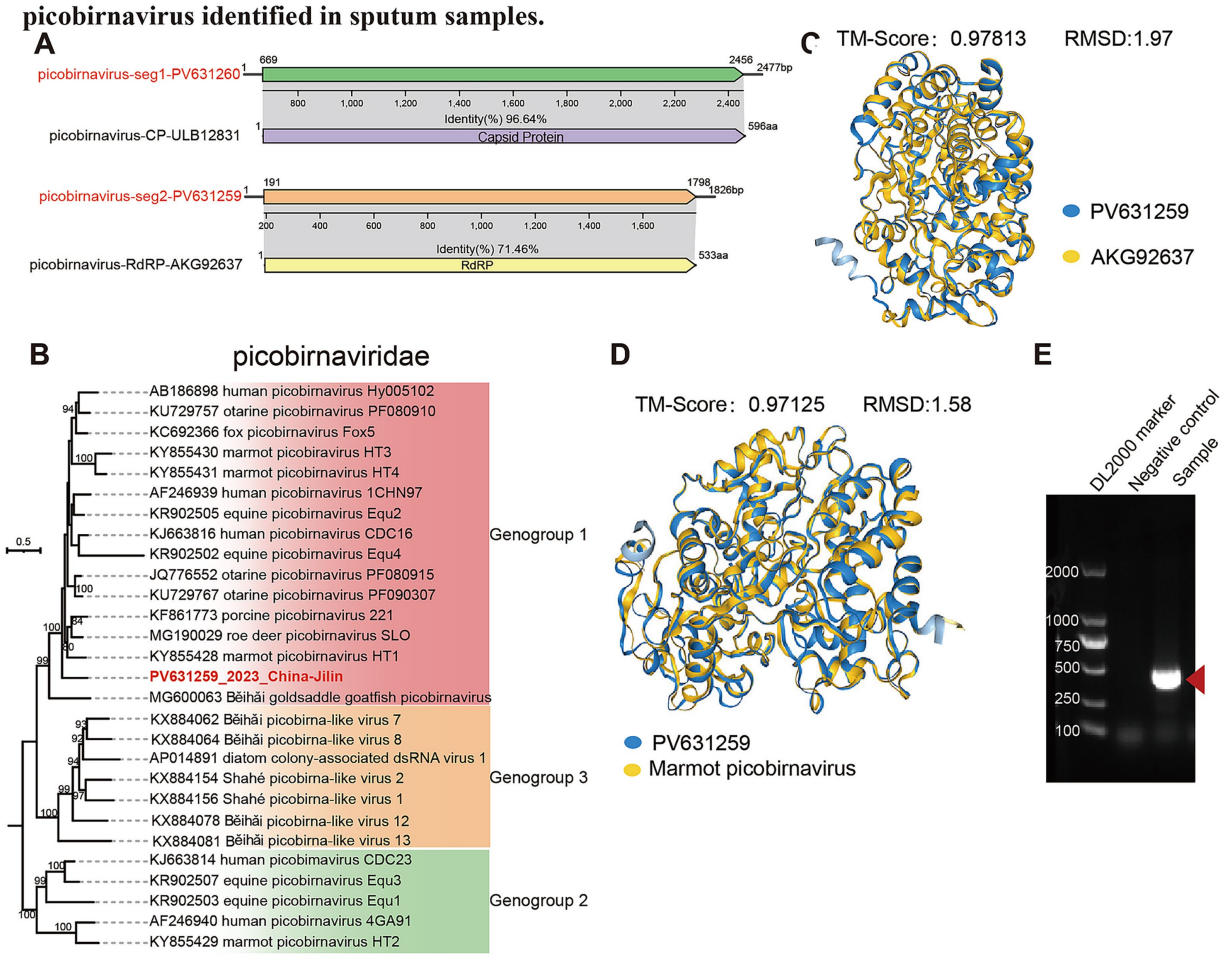
Genomic characterization, phylogenetic tree, structural alignment, and validation of a novel picobirnavirus identified in sputum samples. **(A)** BLASTx analysis of the assembled picobirnavirus genome sequences. The capsid protein encoded by the assembled sequence PV631260 shows the highest amino acid similarity (96.64%) to protein ULB12831. The RdRP encoded by PV631259 exhibits the highest similarity (71.46%) to protein AKG92637. **(B)** Maximum likelihood phylogenetic tree of the family *Picobirnaviridae*. The tree was constructed based on nucleotide sequences encoding the RNA-dependent RNA polymerase (RdRP), following the reference dataset from ICTV. Sequences highlighted in red represent genomes assembled in this study. The picobirnavirus identified here clusters within genogroup 1. **(C)** Structural alignment between picobirnavirus identified in this study (PV631259) and picobirnavirus RdRP AKG92637. **(D)** Structural alignment between the picobirnavirus identified in this study (PV631259) and the RdRP protein of its closest relative in the *Picobirnaviridae* phylogenetic tree, marmot picobirnavirus HT1. **(E)** Gel electrophoresis of the PCR product targeting the novel picobirnavirus from sputum samples. The expected band size was observed, confirming the amplification of the target fragment.

Beyond well-characterized respiratory viruses, this study successfully assembled a novel picobirnavirus from adult sputum samples. The virus exhibits a bipartite genome typical of picobirnaviruses: segment 1 (GenBank accession number: PV631260) encoding the capsid protein(C) and segment 2 (GenBank accession number: PV631259) encoding the RNA-dependent RNA polymerase (RdRp). Comparative genomic analysis using NCBI BLASTn revealed that segment 1 shares 91.93% nucleotide identity and 80% coverage with a previously reported picobirnavirus capsid sequence (GenBank: OL875303.1, derived from sputum in Colombia). In contrast, segment 2 exhibited only 3% coverage but 92.06% identity with a picobirnavirus RdRp sequence from a rodent metagenome in China (GenBank: PQ792594.1) (Table 1). In contrast to the low nucleotide coverage, NCBI BLASTx analysis of PV631259 (segment 2) demonstrated stronger homology, with 88% coverage and 71.46% amino acid identity to the RdRp of a human picobirnavirus (GenBank: AKG92637.1), isolated from upper respiratory swabs in Cambodia. The assembled C protein shares 96.64% amino acid identity and 72% query coverage with a human picobirnavirus reference sequence (GenBank: ULB12831.1, sputum from Colombia), supporting its classification as a human-associated strain (Figure 5A). Phylogenetic analysis based on ICTV criteria further resolved the virus within Genogroup 1 of the *Picobirnaviridae* family, distinct from Genogroups 2 and 3 (Figure 5B).

In addition to sequence-based analyses, we performed three-dimensional structural comparisons of the RdRP protein identified in this study. The structural alignment revealed that the RdRP protein obtained here exhibits a high degree of similarity to the reference sequence AKG92637.1. Specifically, the Foldseek analysis yielded a TM-score of 0.97813 and an RMSD of 1.97 Å, indicating that the two proteins are nearly identical in their overall fold and spatial conformation (Figure 5C). We also ompared the RdRP with other phylogenetically related picobirnaviruses. It showed strong structural similarity to marmot picobirnavirus HT1 (TM-score 0.97125, RMSD 1.58 Å) and Beihai goldsaddle goatfish picobirnavirus (Figure 5D; Supplementary Figure S1A). Within Genogroup 1, it closely matched human strains AF246939, KJ663816, and AB186898, all with TM-scores ≥0.94 and RMSD ≤1.65 Å (S1 B–D). In contrast, comparisons with Genogroup 2 (AF246940, KJ663814) and Genogroup 3 (KX884156) viruses showed markedly lower similarity, with TM-scores of 0.77051, 0.78922, and 0.82161, respectively (Supplementary Figures S1E–G). These results further support that the RdRP protein identified in this study is highly homologous and strongly conserved relative to known picobirnaviruses within genotype1. To validate these findings, nested PCR was performed on adult sputum samples using primers specific to the novel picobirnavirus. Amplicons of the expected size were visualized via gel electrophoresis, and Sanger sequencing confirmed 100% identity between the PCR products and the assembled viral genome, corroborating its presence in clinical samples (Figure 5E).

## Discussion

In this study, we comprehensively characterized the respiratory virome across diverse clinical specimens, including sputum, throat swabs, blood and BALF, collected from northeast China. We successfully assembled complete or near-complete genomes of common respiratory viruses spanning the *Coronaviridae*, *Pneumoviridae*, *Orthomyxoviridae*, and *Picornaviridae* families, alongside HPgV and a novel *picobirnavirus.* To our knowledge, this represents the first report providing full genomic sequences of respiratory viruses from this understudied region, offering critical baseline data for regional virome surveillance. Our findings underscore the extensive circulation of respiratory viruses across heterogeneous populations and specimen types, reinforcing the value of mNGS in uncovering both known and emerging viral diversity.

Yin et al. identified HCoV-229E, HMPV, HRV, IAV, IBV, and HRSV in adult BALF and sputum samples from Jilin province in 2017–2018 (19), aligning with our findings where these viruses were detected and their complete genomes assembled. Notably, influenza viruses, HRSV, and HRV exhibited high reads per million (RPM) in BALF samples, while influenza viruses and HRSV predominated in pediatric throat swabs. The microbiome analysis revealed *Veillonella* as a major bacterial genus, constituting 22.30% of total bacterial reads in BALF samples and 7.7% in pediatric throat swabs. In contrast, *Granulicatella* was absent in BALF and represented only 0.24% of bacterial reads in pediatric samples. This parallels observations by Li et al. (20), who reported significant enrichment of *Veillonella* and minimal *Granulicatella* abundance in patients infected with IV, HRSV, and HRV compared to healthy controls, reinforcing the association between these microbial signatures and respiratory viral infections. The pediatric throat swab and adult sputum samples, which tested positive for IV and HMPV infection, also exhibited a notably high abundance of *Streptococcus*. While previous studies have reported the increased abundance of *Streptococcus* in the nasopharyngeal samples of IV patients, a concurrent decrease has been observed in the oropharyngeal from the same cohort (21). These findings suggest that the relationship between IV infection and *Streptococcus* is complex and may be influenced by sampling site and other contextual factors Further investigation is needed to clarify this relationship and to unravel the intricate interplay between respiratory bacteria and viruses in different anatomical niches.

Our analysis identified HRSV-A, HRSV-B, IAV and IBV in pediatric throat swabs collected during winter in Northeast China. These findings align with surveillance data from 2016 to 2022, which identified HRSV, IAV, and IBV as the predominant respiratory viruses affecting children in this region during winter months (22). Similarly, consistent with a 2014–2016 Chinese study (23), we detected and assembled complete genomes of HRSV, HMPV, IAV and IBV from pediatric samples, underscoring their persistent circulation. HRSV subtypes in China exhibit variable circulation patterns, characterized by alternating dominance cycles (e.g., “ABBAABAABAAABB” from 2008–2021) between HRSV-A and HRSV-B (24). While our study detected both subtypes in respiratory samples, HRSV-A was restricted to pediatric throat swabs, whereas HRSV-B was identified in both adult and pediatric populations. This discrepancy underscores the need for expanded, longitudinal sampling to resolve subtype-specific transmission dynamics and refine our understanding of HRSV epidemiology in China.

HCoV-229E, possessing a long evolutionary history and circulating widely among humans and various animal hosts (25, 26), underwent genotype diversification beginning around 1955. Genotypes 1 through 4 emerged between the 1970s and 1990s (27). While strains detected in Guangdong, southern China (2009) belonged to Genotypes 4 and 5, Genotype 6 subsequently emerged as the dominant global lineage (28). Recently, a distinct emerging lineage was identified in Beijing, China (2019) (29). Consistent with this, the complete HCoV-229E genome assembled in our study, together with recent sequences from the United States (2022, 2023) and Senegal (2022, 2023) (30), clusters genetically with the Beijing strains and is classified within this emerging lineage. This collective evidence indicates that this emerging lineage of HCoV-229E is now established as the dominant lineage circulating globally.

In addition, we assembled three complete genomes of SARS-CoV-2 from pediatric throat swabs, adult sputum, and adult BALF samples. The sequenced genomes correspond to Omicron lineages BA.2, BA.2.86, and HK.3, respectively. These findings align with SARS-CoV-2 genomic surveillance data from Guangzhou (April 2023–March 2024), which identified EG.5.1 and HK.3 as the predominant lineages between epidemiological weeks 27 and 52 of 2023 (31). Globally, descendants of the XBB recombinant lineage, including XBB.1.5, XBB.1.16, and EG.5.1, dominated circulating variants by mid-2023. HK.3, a descendant of XBB, further exemplifies this evolutionary trajectory. Of particular concern is BA.2.86, a highly mutated BA.2 sublineage first detected in July 2023, which has garnered international attention due to its enhanced immune evasion potential (32–34). Collectively, our study not only delineates the diversity of SARS-CoV-2 lineages circulating in Northeast China during winter 2023 but also corroborates national genomic surveillance trends. These results underscore the widespread dissemination and transmissibility of emerging variants such as BA.2.86 and HK.3 across China, highlighting their public health relevance.

In this study, we determined that H3N2 was the predominant influenza subtype during the 2023 winter season, aligning with trends observed in Taiyuan, Shanxi (Northern China) (35). These findings partially contrast with concurrent data from the United States, where pH1N1 and H3N2 co-circulated in Arizona (October 2023–February 2024), but pH1N1 emerged as the dominant strain (36). This highlights persistent regional variations in influenza epidemiology despite increased global travel and viral dissemination. Notably, the two IAV genomes sequenced in this study showed close phylogenetic relatedness to strains from Liao and Heilongjiang provinces, along with a subset of strains identified in Hainan (south of China) (37), all belonging to subclade 3C.2a1b.2a.2a.3a.1. This subclade is corroborated by GISAID data as responsible for the 2023–2024 global epidemic. Thus, the 2023 Jilin IAV strains correspond to contemporaneous global circulation patterns.

Time-dependent genetic evolution was observed for IBV. Strains from China (2018–2019) belonged to lineages within the V1A clade (including V1A, V1A.1, V1A.2, V1A.3), while strains from Guangdong in 2021 were assigned to the V1A.3a.1 subclade (38). By 2023–2024, subclade V1A.3a.2 had become the globally dominant lineage (39). Consistent with this trend, the three near-complete IBV genomes identified in this study also belong to the V1A.3a.2 sublineage and display high sequence similarity with strains from Liaoning province.

HPgV, a virus structurally and epidemiologically related to Hepatitis C virus (HCV), is transmitted primarily through blood or blood products (e.g., needle-sharing, hemodialysis), sexual contact, or vertical mother-to-child transmission. Despite its initial identification in hepatitis patients, HPgV infection exhibits no clear association with chronic liver disease (40). Co-infections of HPgV-1 with HIV-1, HCV, or HBV are frequently observed, while meta-analyses also identified associations between HPgV-1 and non-Hodgkin lymphoma as well as sporadic encephalitis (41). To date, HPgV has been classified into seven genotypes, each with distinct geographic distributions. Feng et al. detected human pegivirus (HPgV) genotype 1 by annotating reads in blood samples from healthy individuals in Beijing, north of China (42), while our study assembled a nearly complete genome of HPgV-3. Historically, genotype 3 has been reported as the dominant HPgV lineage in China (43, 44). However, regional disparities exist: in Yunnan Province, genotype 7 prevails among intravenous drug users (IDUs), whereas genotype 3 remains predominant among men who have sex with men (MSM) in Beijing (45, 46). These observations raise critical questions about whether transmission routes or population-specific risk facttttIn summary, we employed mNGS to comprehensively characterize the respiratory virome in Northeast China. By assembling genomes of common respiratory viruses and reconstructing their phylogenetic relationships, we generated high-resolution insights into viral diversity and evolution in the region. Notably, we identified a novel picobirnavirus in sputum samples, marking the first report of this virus in China. These findings provide critical baseline data and genomic resources to advance regional virome surveillance and inform public health strategies for emerging respiratory pathogens.

## Methods

### Sample collection

Samples in this study were collected in December 2023 at a hospital in Changchun city of Jilin Province, China, following approval by the Medical Ethics Committee of the First Affiliated Hospital of Jilin University (approval number: 2023–661). A total of 445 cases from different individuals, including 95 cases of peripheral blood serum from children, 90 cases of throat swabs from children, 100 cases of peripheral blood plasma from adults, 100 cases of sputum specimens from adults, and 60 cases of bronchoalveolar lavage fluid from adults.

### RNA extraction, library construction and sequencing

Samples were pooled according to their source department of hospital. Next, RNA extraction from each pool was used TRIzol Reagent according to the manufacturer’s instructions. In addition, 2 × 10^5^ of Vero cells were added to each pool to improve the library construction success rate. Meta-transcriptome library preparation of each pool was carried out using the TruSeq™ Stranded Total RNA Sample Preparation Kit from Illumina (San Diego, CA) after removal of host ribosomal RNA using Illumina Ribo-Zero™ rRNA Removal Kits (San Diego, CA). Paired-end (150 bp) sequencing of each RNA library was performed using the Illumina Novaseq 6,000 platform.

### Reads assembly and classification

For each library, reads were first quality controlled to remove adaptor and low-quality reads using Trimmomatic (version 0.39) with the following parameters: SLIDINGWINDOW:4:15 LEADING:3 TRAILING:3 MINLEN:36. Next, human reads, *Chlorocebus sabaeus* reads and ribosomal RNA were excluded with human reference genome (GRCh38.p14), *Chlorocebus sabaeus* genome (GCA_015252025.1) and rRNA contigs downloaded from SILVA database using BBMap (version 38.18) with parameter minid = 0.90,maxindel = 3, bwr = 0.16, bw = 12, minhits = 2, quickmatch fast. Then, Kraken2 (version 2.1.2) and Bracken (version 2.9), using the k2_pluspf_20221209 database, were taken to perform taxonomic classification and abundance estimation on the sequencing reads. The remaining reads were assembled using MEGAHIT (version 1.2.9) with default parameters. To refine incomplete viral genomes, the assembled contigs were subjected to BLASTn, and the viral genome with the highest-identity hit was selected as the reference genome. Sequencing reads were then mapped to the reference genome using BBMap, and the mapped reads were subsequently *de novo* assembled using MEGAHIT (options –k-min 21 –k-max 255 –k-step 10).

### Viral taxonomic classification and abundance

The assembled contigs were subjected to the top Diamond blastx (version 2.1.9.163) hit against the NCBI non-redundant protein database (nr) database. The E-value threshold was set at 1E-5. Then, the contigs taxonomically classified as originating from the kingdom of “Viruses” were identified as probable virus sequence. The remaining reads which exclude host and ribosomal RNA, were mapped to probable virus sequence using Bowtie2 (version 2.4.4). Next, using CheckM (version 1.2.2) to obtained the mapped reads of each sequence by executing the checkm coverage command. The relative abundance was quantified in terms of the number of mapped reads per million of total reads (RPM) in each library. To reduce false-positives, the viral contigs with an RPM < 1 was discarded. CheckV (version 1.0.3) was used to estimate the completeness, contamination of viral sequence.

### Phylogenetic analyses

We conducted a phylogenetic analysis of viruses assembled from this study, to determine their genotypes, assess their prevalence in the northeastern region of China, and examine their phylogenetic relationships with other sequences from China. The complete Chinese sequences of this viral species were downloaded from NCBI and GISAID. Sequences originating from the China or northeastern region of China (IVA) were retained, while the remaining sequences were subjected to redundancy reduction using CD-HIT (version 4.8.1) with the parameter (−c ≥ 0.988). Overall, the dataset for phylogenetic analysis was composed of assembled viral sequences, globally representative reference sequences downloaded from NCBI and GISAID, and the redundancy-reduced Chinese sequences. The sequence dataset for each virus was aligned using MAFFT (version 7.490) for multiple sequence alignment, with ambiguous regions removed using trimAL (version 1.5). The phylogenetic tree was constructed using the maximum likelihood method with IQ-TREE (version 2.0.7) employing the best-fit substitution model selected by the setting “-m MFP,” the ultrafast bootstrap parameter was set to 10,000, and all other parameters left at their default settings. All resulting treefiles was visualized using iTol (v6.9; https://itol.embl.de/). For SARS-CoV-2, the phylogenetic clades of each sequence and its corresponding Pango lineage was determined using the Pangolin (Phylogenetic Assignment of Named Global Outbreak Lineages) web server v4.3 (https://pangolin.cog-uk.io/, accessed on 28 November 2024).

### Confirmation of the novel picobirnavirus

To confirm the presence of the newly detected *picobirnavirus*, virus-specific primers were designed based on the assembled viral contigs for nested PCR assays (Table S2). The PCR products matching the expected target size were further validated by Sanger sequencing.

### Mutation analysis of the SARS-CoV-2 spike protein

Amino acid mutations in the spike proteins of all sequences included in the SARS-CoV-2 phylogenetic tree were analyzed using Nextclade Webserver v3.18.0 (accessed November 2025). The Nextclade server used, by default, Wuhan-Hu-1/2019 (MN908947) as the reference sequence. The top 50 spike protein mutations were visualized using the ggplot2 package in R (4.3.0).

### Structural alignment of picobirnavirus proteins

The picobirnavirus genomes assembled in this study were annotated using Prokka (version 1.14.6), and the predicted coding sequences were translated into amino acid sequences. Protein structures were subsequently predicted with the AlphaFold server (https://alphafoldserver.com/). Structural comparisons of the viral proteins were then performed using the Foldseek (version 9.427df8a) easy-search workflow.

## Data Availability

The datasets presented in this study can be found in online repositories. The names of the repository/repositories and accession number(s) can be found in the article/Supplementary material.
