# Correction: Genetic characterization of the respiratory tract viruses in Jilin, Northeast China, 2023

**DOI:** 10.3389/fpubh.2026.1788645

**Published:** 2026-01-29

**Authors:** Zhixia Song, Yuanyuan Huang, Yue Gu, Lihe Che, Kaiyu Zhang, Quan Liu, Qingtian Guan, Liyan Sui

**Affiliations:** 1 State Key Laboratory for Diagnosis and Treatment of Severe Zoonotic Infectious Diseases, Department of Infectious Diseases and Center of Infectious Diseases and Pathogen Biology, Key Laboratory of Organ Regeneration and Transplantation of the Ministry of Education, The First Hospital of Jilin University, Changchun, China; 2 Department of Paediatrics, First Hospital of Jilin University, Changchun, China; 3 Department of Respiratory and Critical Care Medicine, First Hospital of Jilin University, Changchun, China; 4 Bioinformatics Laboratory, Infectious Diseases and Pathogen Biology Center, The First Hospital of Jilin University, Changchun, China

**Keywords:** human pegivirus, metagenomic sequencing, novel picobirnaviru*s*, phylogenetic analyses, respiratory viruses

An incorrect **Funding** statement was provided. The correct funding statement reads:

“The author(s) declared that financial support was received for this work and/or its publication. This work was supported by the National Natural Science Foundation of China grants 82341105 and 82372250 (QL), Jilin Provincial Science and Technology Development Project grant number YDZJ202401276ZYTS (QG), Start-up Research Funding 04045970001 (QG), Jilin Province Health Talent Construction Project grant J1SRCZX2025–127 (LS), and Norman Bethune Plan Project of Jilin University grant 2024B29 (LS).”

The original version of this article has been updated.

