# Supplementary material for: Genetic characterization of the respiratory tract viruses in Jilin, Northeast China, 2023
Source: Front Public Health. 2026 Jan 13;13:1756127. doi: 10.3389/fpubh.2025.1756127 (PMC12836386; doi:10.3389/fpubh.2025.1756127)
Supplement: Supplementary file 1 [file Data_Sheet_1.PDF]

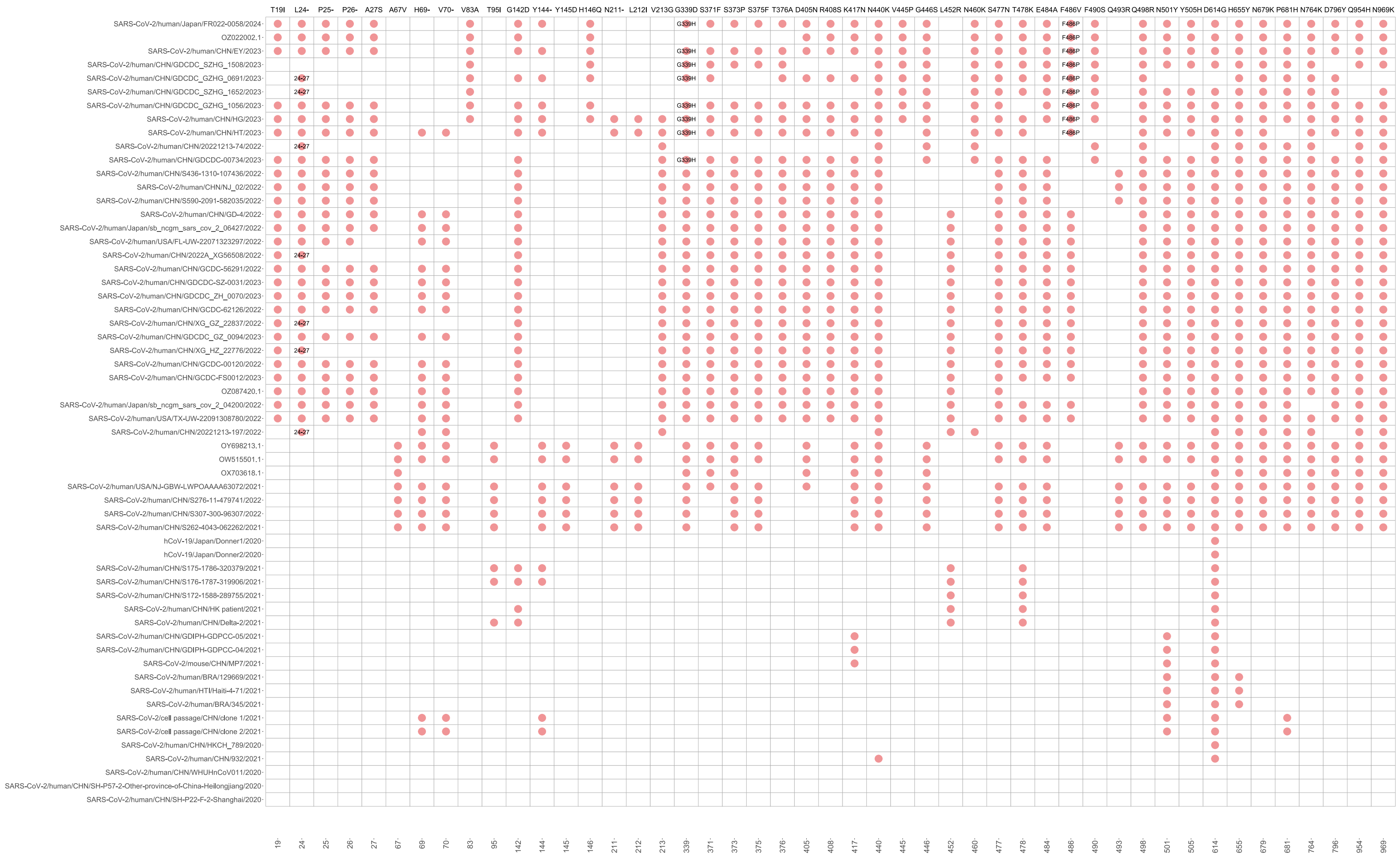

S1. Top 50 mutations in the S protein across all strains in the phylogenetic tree.

Major mutations at each position are shown at the top, whereas minor, less frequent mutations are displayed within the corresponding cells.

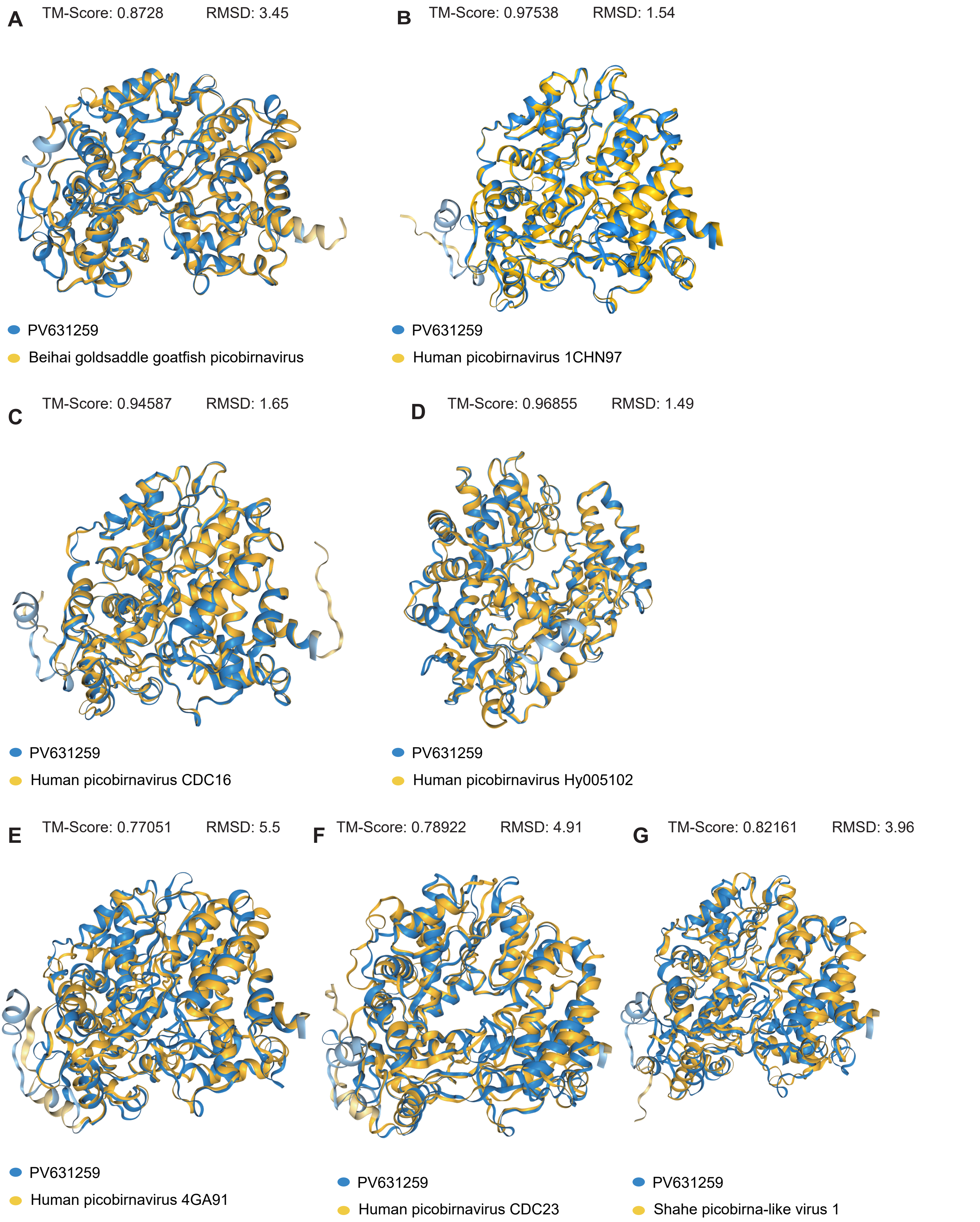

S2. Structural alignment of the RdRP protein of the novel picobirnavirus identified in this study with sequences from known *Picobirnaviridae* strains or metagenome-assembled virus.

Structural alignment between the picobirnavirus identified in this study (PV631259) and the RdRP proteins of: (A) its closest relative in the Picobirnaviridae phylogenetic tree, Beihai goldsaddle goatfish picobirnavirus; Genogroup 1 viruses: (B) human picobirnavirus 1CHN97, (C) human picobirnavirus CDC16, and (D) the representative human strain AB186898 human picobirnavirus Hy005102; Genogroup 2 viruses: (E) human picobirnavirus 4GA91 and (F) human picobirnavirus CDC23; and (G) the Genogroup 3 virus Shahe picobirna-like virus 1.
