# Supplementary material for: Genetic characterization of the respiratory tract viruses in Jilin, Northeast China, 2023
Source: Front Public Health. 2026 Jan 13;13:1756127. doi: 10.3389/fpubh.2025.1756127 (PMC12836386; doi:10.3389/fpubh.2025.1756127)
Supplement: Supplementary file 2 [file Data_Sheet_2.PDF]

Supplementary material

Characterization of the respiratory tract viruses in Jilin, northeast China, 2023

Table S1. Other virus segments from the RNA sequencing libraries.

Viral sequences identified in samples	Consensus sequence RNA library	Query cover	Nucleotide identity to closest sequence	Closest sequence accession number	Sequence length	Assembly length	Assembly accession number
Influenza A virus segment1	HT	92%	99.83%	PP875527.1	2316	2312	PV571918
Influenza A virus segment1	EY	100%	99.73%	OR010104.1	2316	2255	PV571917
Influenza A virus segment2	EY	100%	99.78%	OR012086.1	2316	1792	PV571912
Influenza A virus segment3	HT	69%	100%	PP688196.1	2208	2221	PV571909
Influenza A virus segment3	EY	99%	99.71%	PP402261.1	2151	1026	PV571906
Influenza A virus segment5	HT	100%	100%	PP683588.1	1540	1535	PV571910
Influenza A virus segment5	EY	100%	99.67%	OR014265.1	1542	1493	PV571914
Influenza A virus segment6	HT	100%	100%	PQ578516.1	1440	1404	PV571915
Influenza A virus segment6	EY	100%	99.71%	OR010109.1	1441	1386	PV571908
Influenza A virus segment7	EY	100%	99.80%	PQ578505.1	1002	992	PV571913
Influenza A virus segment8	HT	100%	100%	PP332072.1	865	856	PV571907
Influenza B virus segment 1	HG	99%	99.96%	PQ069971.1	2324	2354	PV597936

Influenza B virus segment 1	HT	100%	99.96%	PP645076.1	2340	2340	PV597948
Influenza B virus segment 1	EY	100%	99.91%	PQ069971.1	2324	2255	PV597932
Influenza B virus segment 2	HT	92%	100%	PP645075.1	2367	2570	PV597946
Influenza B virus segment 2	HG	98%	99.96%	PP645075.1	2367	2425	PV597943
Influenza B virus segment 2	EY	100%	99.91%	PP535413.1	2367	2299	PV597933
Influenza B virus segment 3	HG	100%	99.87%	PQ253201.1	2275	2284	PV597938
Influenza B virus segment 3	HT	100%	99.78%	PP645077.1	2275	2271	PV597949
Influenza B virus segment 5	HT	96%	99.89%	PP645079.1	1815	1813	PV597945
Influenza B virus segment 5	EY	100%	99.83%	OR629124.1	1815	1767	PV597935
Influenza B virus segment 5	HG	100%	99.78%	PP535425.1	1815	1817	PV597940
Influenza B virus segment 6	HG	100%	99.93%	PQ069916.1	1514	1503	PV597939
Influenza B virus segment 6	HT	93%	99.80%	PP594118.1	1532	1614	PV597947
Influenza B virus segment 6	EY	100%	99.72%	OR629437.1	1528	1435	PV597934
Influenza B virus segment 7	HT	94%	100%	PP645081.1	1154	1184	PV597951
Influenza B virus segment 7	HG	96%	99.91%	PP535407.1	1154	1158	PV597942
Influenza B virus segment 7	EY	99%	99.73%	PP535407.1	1154	1133	PV628740
Influenza B virus segment 8	EY	100%	100%	PP642051.1	1066	1030	PV597930

Influenza B virus segment 8	HG	99%	100%	OY283099.1	1066	1082	PV597937
Influenza B virus segment 8	HT	99%	99.72%	OY283099.1	1066	1117	PV597944

Table S2. The nested PCR primers used to confirm the novel picobirnavirus

Primer name	Primer sequence (5' - 3')	Length pf product(bp)
Pico-692F1	AGAGATGGCCTTACATACTCG	
Pico-713F2	GACATTAAGGGCAAACTCT	503
Pico-1196R	TAATGCCCTATGTAGTAGTGT	
